# Immunotherapeutic advances in gastrointestinal malignancies

**DOI:** 10.1038/s41698-018-0076-8

**Published:** 2019-02-05

**Authors:** Devika Rao, Ruwan Parakrama, Titto Augustine, Qiang Liu, Sanjay Goel, Radhashree Maitra

**Affiliations:** 1 Montefiore Medical Center, 1695 Eastchester Road Bronx, New York, 10461 USA; 2 Albert Einstein College of Medicine, 1300 Morris Park Ave Channin 302D, Bronx, NY 10461 USA; 3 Department of Pathology Montefiore Medical Center, 111 E. 210th, St. Bronx, NY 10467 USA; 4 Department of Biology, Yeshiva University, 500W 185th Street, New York, NY 10033 USA

## Abstract

Cancer is an important global issue with increasing incidence and mortality, placing a substantial burden on the healthcare system. Colorectal cancer is the third most common cancer diagnosed among men and women in US. It is estimated that in 2018 there will be 319,160 new diagnosis and 160,820 deaths related to cancer of the digestive system including both genders in the United States alone. Considering limited success of chemotherapy, radiotherapy, and surgery in treatment of these cancer patients, new therapeutic avenues are under constant investigation. Therapy options have consistently moved away from typical cytotoxic chemotherapy where patients with a given type and stage of the disease were treated similarly, to an individualized approach where a tumor is defined by its specific tissue characteristics /epigenetic profile, protein expression and genetic mutations. This review takes a deeper look at the immune-biological aspects of cancers in the gastrointestinal tract (entire digestive tract extending from esophagus/stomach to rectum, including pancreatico-biliary apparatus) and discusses the different treatment modalities that are available or being developed to target the immune system for better disease outcome.

## Introduction

A deeper understanding of the biology driving cancer has helped shape treatment approaches. Cancer therapy options have consistently moved away from typical cytotoxic chemotherapy where patients with a given cancer were treated equal, to an individualized approach where a tumor is defined by its genetic profile, pertaining to protein expression and gene mutations. The latest addition to the treatment arsenal is immunotherapy, where the patient’s own immune system is reprogrammed to recognize and target the tumor.

**Fig. 1:**
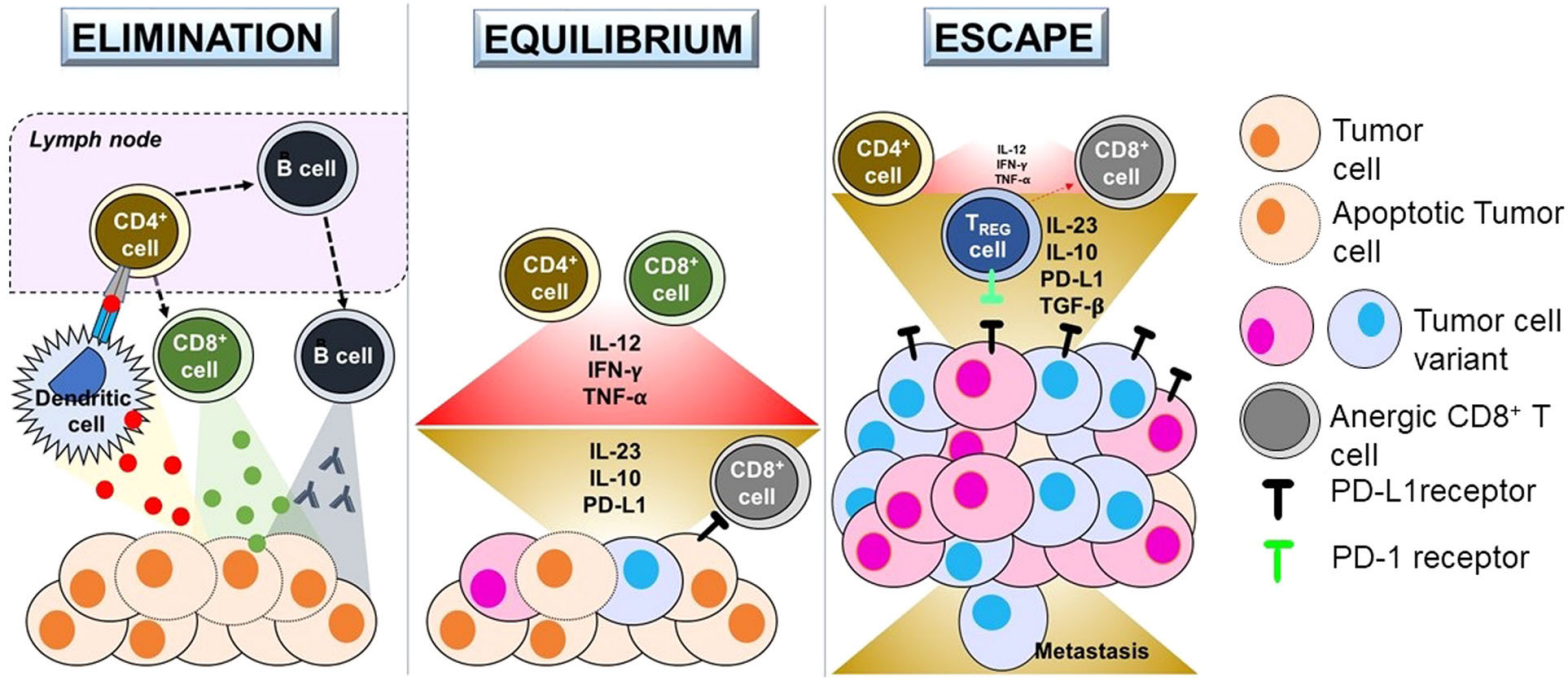
Elimination—(1) Apoptotic tumor cells release antigens which are collected by Dendritic cells, (2) Dendritic cells present antigen to CD4 + T cells in lymph node, which leads to the activation of cytotoxic CD8 + T cells and B cells, (3) B cells release antibodies; CD8 + cells release and Perforin/Granzyme, resulting in tumor destruction. Equilibrium—Immune system keeps the tumor in a state of dormancy. Anti-tumor cytokines (IL-12, IFN-γ, TNF-α) and cytotoxic action is countered by pro-tumorigenic/anergy-inducing molecules (IL-10, IL-23, PD-L1) from the tumor. Alteration of genetic pathways within tumor cells also generates new variants which can avoid detection. Escape—Tumor variants utilize (1) decreased expression of antigenic cell surface markers, (2) increased expression of T-cell anergy-inducing cell surface markers (PD-L1, CTLA4), as well as (3) TREG inhibition (via PD-1/PD-L1 interaction) of CD8 + T cells to overpower immune system. Steps (1), (2) and (3) ultimately result in growth, metastasis, angiogenesis and clinical presentation

The relationship between immunology and cancer dates to the late 19th century. One of the first observation documented that an injection of heat-inactivated bacteria into sites of sarcoma sometimes lead to durable regression.^1,2^ Since then, an impressive amount of research has established that not only does the immune system provide initial identification and targeting, it also continues to protect against any residual or new cancer, engaging in a molecular game of “hide and seek” within the tumor microenvironment in a dynamic process now termed “cancer immunoediting.^3^ This process essentially includes three phases: Elimination (initial response of immune system to tumor), Equilibration (immune-mediated tumor dormancy) and Escape (tumor evasion of immune response) phases (Fig. 1).

## Elimination phase

In the Elimination phase, the adaptive and innate branches of the immune system identify tumor-specific antigens as non-self and target the tumor cell for destruction. Important effector molecules of the former include T cells, important subtypes being CD8^+^ (cytotoxic), regulatory (T_reg_) and CD4^+^ (helper cells); Natural Killer (NK) cells, Antigen Presenting Cells (APCs), the macrophages and dendritic cells (DCs). Activation of T cells requires the presentation of tumor antigen by APCs, the most potent of which are DCs. Antigen presented by DCs on MHC Class I or Class II molecules are recognized by T cell receptors; CD8^+^ and CD4^+^, respectively.^4^ This results in secretion of anti-tumor cytokines namely Type I (IFN-α/β) and II (IFN-γ) interferons, interleukins (IL-12, IL-6) and chemokines (CCL2), which aids the destruction of the tumor cell.^5^ Type I interferons have been shown to be critical for the early activation of the antitumor response, by facilitating the cross-presentation of tumor antigens from CD8α^+^/CD103^+^ DCs to CD8^+^ T cells.^6,7^ Type I interferons are also thought to directly induce apoptotic and anti-proliferative responses in tumor cells, further supporting tumor suppression.^8^

Unlike T cells, NK cells do not require antigen presentation by MHC proteins. Rather, NK cells are recruited to the tumor site by the latter’s expression profile of interleukins and chemokines.^9^ NK cells were shown to eradicate senescent tumor cells in a p53-dependent manner.^10^ Another key pathway to the innate immune response in the Elimination phase is the stimulator of IFN genes (STING) pathway of cytosolic DNA sensing.^8^ Phosphorylation of STING by TRAF2 (TNF receptor associated factor-2)-binding kinase results in the binding, subsequent phosphorylation and release to the nucleus of IFN regulatory factor 3 (IRF3, a transcription factor), which then drives transcription of IFN-β. In the tumor microenvironment, IFN-β leads to the spontaneous generation of antitumor CD8^+^ responses and is critical for T cell priming. Intratumoral delivery of STING agonists is currently being explored. It should be noted that the STING pathway has also been demonstrated to be tumorigenic,^11^ and an optimal therapeutic level of activation is yet to be determined.

## Equilibration phase

The molecular interactions that comprise the Equilibration phase have not yet been fully discerned, due to lack of appropriate animal model. However, evidence supporting the existence of this phase, as well as potential key interactions between the immune response and tumor do exist. In a mouse model of methylcholanthrene (MCA)-induced fibrosarcoma and p53 mutant tumors, it was shown that: (1) a Th1-like adaptive immune response facilitated tumor dormancy, (2) this dormancy may be a prolonged process, and (3) the balance between IL-12 (anti-tumorigenic) and IL-23 (pro-tumorigenic) in the tumor microenvironment is a determinant in whether the tumor dormancy is achieved.^12,13^ Another study in mice with islet adenomas demonstrated that transplantation of IFN-γ producing, T-cell-antigen-specific Th1 cells inhibited angiogenesis and multi-stage carcinogenesis without tumor cell destruction.^14^ It was subsequently shown that this senescence was mediated by IFN-γ and TNF, by causing permanent growth arrest in the G_1_/G_0_ phase, activation of p16INK4a the inhibitor of cyclin-dependent kinases 4 and 6 and downstream hypophosphorylation of retinoblastoma (Rb) protein.^15^ These data strongly support an immune-mediated mechanism whereby tumors which survive the elimination phase are prevented from reaching full carcinogenic potential. Sadly, this is the proverbial “last stand” of the immune system, as the next phase of tumor development is escape.

## Escape phase

In this phase, the tumor becomes clinically apparent. Mechanisms employed by the tumor in this phase can be distilled into the following three categories:^16^ (1) reduced immune recognition and immune cell stimulation by downregulation of tumor antigens, antigen-expression machinery or co-stimulatory signals—all required for successful activation of APCs and thus T-cells, (2) upregulation of resistance against cytotoxic immunity or upregulation of pro-tumorigenic genes (e.g., STAT3 or Bcl2, respectively), and (3) creation of an immunosuppressive microenvironment.

The generation of an immunosuppressive microenvironment involves several tumors mediated cellular events. In addition to the production of cytokines like VEGF and metabolic factors like adenosine, PGE2, the tumor utilizes the recruitment of TREG cells or myeloid-derived suppressor cells (MDSCs), as well as the ligation of inhibitory receptors (e.g., CTLA-4, PD-1, Tim-3) on immune effector cells to generate adaptive immune resistance. TREG cells express the transcription factor forkhead box-P3 (FoxP3) and are the subset of T cells that suppress the activation, proliferation and effector functions of a wide range of immune cells.^17^ There is evidence that due to the increased metabolic demands of the tumor microenvironment (the “Warburg effect”), tumor-infiltrating lymphocytes are directed towards the expansion of Tregs cells; glucose depletion ultimately inhibits adequate CD8^+^ and CD4^+^ T-cell control of tumor growth.^18^

## Immune interactions in the gut

The gastrointestinal system encounters the largest microbial activity in the body and thus requires multiple protective mechanisms to counter invasion by exogenous and endogenous/commensal microbes, as well as various infectious agents (e.g., viruses, parasites). Thankfully, mucosal immunity is a well-functioning, coordinated surveillance system, interwoven with the physiological and mechanical alterations that comprise gut homeostasis.^19^ Innately, intestinal epithelial cells (IECs); absorptive epithelial cells, goblet cells and Paneth cells provide physical and chemical barriers to infection, by the secretion of mucus and anti-microbial peptide (AMP), respectively. IECs present antigens to dendritic cells and macrophages, and directly modulate immune cell responses via secretion of cytokines and chemokines.^20,21^ Additionally, the intestinal lamina propria contains a variety of myeloid and lymphoid cells, which can communicate to one another via direct contact, or by using cytokine signaling.^22^ The immunological diversity here includes CD4^+^ memory and effector (T helper 1 (Th1), Th17) T cells, and regulatory (T_reg_) T cells. T_reg_ cells play a key role in suppressing inflammation, via the secretion of IL-10. Myeloid derivatives include dendritic cells and macrophages – subtypes of the latter have been shown to secrete IL-10 in response to commensal bacteria via the Toll-like receptor (TLR) signaling pathway, preventing inflammation.^23^ Conversely, immune cells can also interact with IECs. Th17 cells secrete IL-22, which upregulates secretion of AMP.^24^ IL-6 production from IEC lymphocytes facilitates proliferation of the intestinal epithelium, promoting healing after mucosal injury.^25^

Mucosal immunity thus tightly regulates inflammation in the gut. This regulation is critical, as an unchecked and/or prolonged inflammatory state has been recognized as a potential driver for the development of colorectal cancer (CRC), the third most frequent cause of cancer related mortality amongst men and women in the US. The current understanding of tumor progression in CRC revolves around the initiation and maintenance of non-specific inflammation, which results in the production of pro-inflammatory cytokines (e.g., TNF-α, IL-1β). These cytokines can act directly on IECs, promoting proliferation, inhibition of apoptosis, invasion, angiogenesis, epithelial to mesenchymal (EMT) transition and metastasis.^26^ Additionally, anti-tumor (GM-CSF, IFN-γ) cytokines are depleted in CRC,^27^ which not only accelerates tumor progression but also results in poorer outcomes for patients.

## Gut Microbiome

In the past decade, as therapy options have expanded, we are learning to appreciate and possibly target this tumor microenvironment where different inflammatory cells and mediators co-exist in a state of delicate balance. The microenvironment in the gut includes over 1100 prevalent species and at least 160 species of bacteria, archaea, microeukaryotes, and viruses per individual.^28^ The gut microbiome appears to remain relatively resilient over time, and studies have indicated that certain microorganisms have the ability to influence and enhance metabolism, the immune system, cancer resistance, endocrine signaling, and brain function.^29^ Instability in the composition of gut bacteria (dysbiosis) has been linked to common human intestinal disorders, such as Inflammatory Bowel Disease (IBD) and a few cancers such as gastric cancer. While the role of *H. pylori* in gastric cancers is well-established,^30^ the role of the microbiome in CRC is still unclear. Studies in mouse models of altered immune and inflammatory responses suggest that dysbiosis could be sufficient to promote cancer.^31,32^ This gains clinical significance, as it provides a novel avenue for therapeutic targeting. Two clinical studies of CRC patients in China showed that oral administration of *Bifidobacterium* or intestinal probiotics tablets improved bacterial dysbiosis and immunity.^33^ *Lactobacillus*, which is high in probiotics, has been shown to have anti-CRC effect in vitro and vivo studies.^34–36^ Another avenue under exploration is the Fecal Microbiome Transplant (FMT), which has shown potential in treatment of infections such as *C.difficile* and IBD. However, the mechanisms that contribute to dysbiosis are not completely understood and whether dysbiosis is a cause or an effect of CRC is still under scrutiny.

The role of the microbiome is further highlighted by its influence on the efficacy of immune-mediated therapies. The first preclinical studies looking at the changes in the gut microbiota in mice when exposed to the anti-PD-1/anti-CTLA4 agents was conducted in 2015.^37^ The efficacy of anti-CTLA4 therapy was significant decreased in mice treated with antibiotics, with a particular increase in *Bacteroidales* and *Burkholderiales* as favored species. This knowledge was further explored in humans and multiple studies substantiating this finding have been reported.^38,39^ Studies have shown that patients who were pretreated with antibiotics for routine indications, prior to immunotherapy, had a diminished response in terms of a lower progression-free survival and overall survival rates compared with patients who had not received antibiotics. Whether supplementing certain species of organisms through FMT can help increase/synergize response to immunotherapy is still under evaluation and is an important avenue for future research.

## Immunoscore

With increasing importance given to immune based therapy, there is a growing sentiment that classification systems such as the traditional American Joint Committee on Cancer/Union Internationale Contre le Cancer (AJCC/UICC) TNM staging system provide insufficient prognostic information.^40^ Two alternate markers that have gained significant importance with the changing landscape of therapy are as follows (a) status of microsatellite instability (MSI) identified through molecular genetics and (b) host immune infiltration of tumors on immunohistochemistry analysis.^7^ Multiple studies have demonstrated that tumors with elevated levels of tumor-infiltrating lymphocytes (TILs) typically have a prominent level of microsatellite instability (MSI-H). Since cancer immunotherapy works by modulation of immune responses in favor of enhancing tumor cell detection and immune clearance of these cells, presence of TILs serves to predict response to immune intervention. This has led to the development of “immunoscore”, a classification system shown to have a prognostic significance superior to that of the AJCC/UICC TNM classification system.^7^ This scoring system has further garnered significance with the introduction of immune checkpoint inhibitors.

## Immune checkpoint inhibitors

Immune “checkpoints” are inhibitory pathways which help differentiate self-antigens from foreign and suppress uncontrolled auto-immunity. Tumor cells evade these checkpoints by genetic and epigenetic alterations to influence neoantigen formation, presentation, and/or processing, as well as alterations in cellular signaling pathways that disrupt the action of cytotoxic T cells.^41^ Identification of the PD-1/PD-L1 and CTLA4 pathways have provided opportunity for manipulation of these checkpoints to block the immune evasion by cancer cells. This new class of drugs allows the host to mount a robust immune response to tumor cells, with scope for long lasting immunity by allowing cytotoxic T cells to recognize tumor antigens and subsequently generating memory T cells. Since the first approval of Ipilimumab for melanoma in 2011, this class of drugs has seen tremendous growth. Due to excellent tumor responses, limited side effect profile and efficacy in numerous solid organ tumors that are otherwise difficult to treat, these drugs have quickly proven themselves to be superior to many cytotoxic chemotherapy regimens.

## Colorectal cancer

Pembrolizumab was the first checkpoint inhibitor targeting the PD-1 pathway to demonstrate clinical activity in solid tumors, including CRC and gastric cancer with micro-satellite instability (MSI). Results from Keynote- 059 led the FDA to grant an accelerated approval to pembrolizumab as treatment for patients without other options with unresectable or metastatic, MSI-H or mismatch repair deficient (dMMR) solid tumors. This was the first approval of a drug for a tissue agnostic indication and this has further paved the path for other clinical trials with umbrella or basket designs. The immune-related objective response rate and immune-related progression-free survival rate were 40% (4 of 10 patients) and 78% (7 of 9 patients), respectively, for dMMR CRC and 0% (0 of 18 patients) and 11% (2 of 18 patients) for pMMR CRC.^42^

There are many theories as to why checkpoint blockers are not efficient in subjects with pMMR CRC. In CRC lesions that are largely infiltrated by effector memory T cell, immunological checkpoints might be intrinsically inactive. In this case, the exogenous administration of checkpoint blockers would be ineffective. Contrarily, CRC lesions with limited T-cell infiltration may not respond to checkpoint blockers because they cannot be properly invaded, recognized or eliminated by the cellular immune system. This may reflect the antigenic properties of malignant cells, their inability to adequately activate the immune system, or the activation of yet to be discovered immunological checkpoints that actively suppress immunosurveillance against CRC.^10^

On the basis of the promising efficacy from pembrolizumab for MSI-H colorectal cancer, it is now understood that somatic mutations have the potential to encode non-self-immunogenic antigens, making these otherwise non-responsive cancers, targetable by immune-mediated intervention. Checkmate 142 is an ongoing clinical trial (NCT02060188) investigating nivolumab in MSI-H metastatic or recurrent CRC, in combination with other checkpoint inhibitors with targets other than the PD-1/PD-L1 axis. The trial is designed to test the efficacy of nivolumab initially in dose escalation (completed) and then in combination with other drugs such as ipilimumab, cobimetinib and daratumumab (ongoing). Among pts treated with nivolumab 3 mg/kg Q2W (N = 74) OS rates were 83.4% (6 mo) and 73.8% (12 mo).^43^ Data from the above study is still maturing and the first full report on the nivolumab + ipilimumab cohort was presented at the ASCO GI Symposium in Jan 2018.^44^ Of 119 treated pts, 76% had ≥ 2 prior lines of therapy. Median follow-up was 13.4 months. The Objective Response Rate was 55% and Disease Control Rate was 80%. Combined treatment with nivolumab/ipilimumab also provided impressive benefits in progression-free and overall survival, with rates at 1 year of 71 and 85%, respectively. Responses were observed regardless of tumor programmed death-1 ligand 1 (PD-L1) expression level or BRAF or KRAS mutation status and were observed in pts with or without a history of Lynch syndrome. Data from this trial has led to the FDA approval of the combination for treatment of MSI-H CRC that has progressed through other cytotoxic chemotherapy regimens.

As our understanding of the molecular mechanisms driving response to immunotherapy improve, trials are being designed with genetically defined patient selection criteria. Additionally, these agents are being tried in combination with other biologics to enhance efficacy. In the case of avelumab, an active trial, NCT03150706, is evaluating use of this drug in patients with MSI-H or POLE mutated CRC. The POLE gene encodes the catalytic subunit of DNA polymerase epsilon, and it involves DNA repair and chromosomal replication. These mutations represent high somatic mutation loads in patients with colorectal cancer, especially in those with MSS. Therefore, MSS tumors harboring POLE mutations might be susceptible to immune checkpoint blockade. Another study, the phase II AVETUX-CRC trial (AIO KRK 0216), is evaluating the feasibility and early efficacy of FOLFOX and cetuximab combined with avelumab in 1st line MCRC.^45^

As we count successes, it is important to remember that only 5% of CRC are MSI-H, with 95% being MSS. As discussed earlier, these cancers have lower immune stimulation and hence poor response to immunotherapy. However, preclinical evidence suggests that inhibition of other pathways such as MEK with cobimetinib leads to upregulation of MHC1 on tumor cells, induces intratumoral T-cell activation, and enhances anti-PD-L1 activity, making the thus far resistant tumors sensitive to immune intervention. This theory is further validated by a phase Ib trial which evaluated synergistic combination of comibetinib and atezolizumab (PD-L1 inhibitor) in several solid tumor types, including heavily pretreated, advanced CRC. Results presented at the 2018 American Society of Clinical Oncology (ASCO) Gastrointestinal Cancers Symposium, show a 6-month progression-free survival (PFS) rate of 18% and a 12-month overall survival (OS) rate of 43%. In the subset of patients with MSS disease, 6-month PFS was 27% and 12-month OS was 51%. The 12-month OS rates compare favorably with the 12-month OS of 24% with regorafenib, a standard treatment option in this setting.^46^

## Hepatocellular cancer

The checkpoint inhibitors have brought about a revolution in treatment of cancers, which were thus far considered to have poor prognosis. This is particularly true in hepatocellular cancers (HCC), where very few drugs have shown efficacy, modest at best, in the past. Checkmate 040, testing Nivolumab in histologically confirmed advanced hepatocellular carcinoma with or without hepatitis C or B has changed this dismal outlook. The objective response rate was 20% (95% CI 15–26) in patients treated with nivolumab 3 mg/kg in the dose-expansion phase and 15% (95% CI 6–28) in the dose-escalation phase. The durable objective responses show the potential of nivolumab in the treatment of advanced hepatocellular carcinoma^47^ and led to its accelerated approval by the FDA. 2018 has seen another accelerated approval of combination therapy with atezolizumab and bevacizumab for patients with advanced HCC. This is based on data from a phase I study presented at the ASCO 2018 meeting in Chicago. Response rate of 61% was observed with median progression-free survival, duration of response, time to progression, and overall survival not yet reached after a median follow-up of 10.3 months.^48^ It is heartening to see multiple drugs approved with durable responses in cancers where there have been no new successful agents for over 10 years (Sorafenib being approved in 2006).

## Gastric and GEJ cancer

While successes have been noted, there have also been some checkpoint inhibitors which have failed to show benefit in large phase III trials. One such example is avelumab, an anti-PD-L1 agent, which initially showed promise in phase Ib trials in patients with gastric and gastroesophageal junction (GEJ) cancers. Findings, announced in Nov 2017, from the phase III JAVELIN Gastric 300 trial, indicate that survival was not improved with avelumab compared with chemotherapy in previously treated patients with gastric or GEJ adenocarcinoma.^49^ A promising study evaluating combination immunotherapy in GEJ and Gastric cancers, was presented at the same ASCO GI Symposium in early 2018. The drugs studied in combination were durvalumab (PD-L1 inhibitor) and ramucirumab (VEGFR2 inhibitor), based on preclinical data suggesting that blocking VEGFR-2 and the PD-1/PD-L1 pathway induces synergic antitumor effects. This promotes access of cytotoxic T cells to tumors, while avoiding the exhaustion of T cells. Preliminary data presented to evaluate safety indicates that the combination generated no unexpected toxicities and demonstrated antitumor activity in patients with previously treated advanced G/GEJ adenocarcinoma. Median PFS was 2.6 months (95% CI, 1.45–6.28).^50^

Currently FDA approved checkpoint inhibitors in GI malignancies, along with indication for use and toxicity profile are listed in Table 1.

**Table 1:** Compilation of approved checkpoint inhibitors in gastrointestinal malignancies along with the target, the year of approval, the specific trial name, benefits, line of therapy and toxicities observed

Cancer type	Target	Drug	Year	Trial	Comparator arm	Benefit observed	Line of therapy	Toxicities of clinical interest	Ref
Colo-rectal	PD-1	Pembrolizumab	2015		MSS tumor	ORR: 40% v 0% PFS: 78% v 11%	MSI-H, unresectable or metastatic	Rash or pruritus (24%); thyroiditis, hypothyroidism, or hypophysitis (10%); and asymptomatic pancreatitis (15%)	^42^
		Nivolumab	2017	CheckMate 142	NA	ORR: 31.1% DCR: 69%	MSI-H, recurrent or metastatic	Arenal insufficiency, transaminitis, colitis, diarrhea, gastritis, stomatitis, acute kidney injury, pain, and arthritis (1 each)	^43, 73^
	CTLA4	Ipilimumab (in combination with Nivolumab)	2018	CheckMate 142	NA	ORR:55% DCR: 80%	MSI-H, progressed on cytotoxic chemo	Transaminitis (11%), elevated lipase (4%), anemia (3%), colitis (3%), diarrhea (22%), fatigue (18%), and pruritus (17%)	^74^
Hepatocellular	PD-1	Nivolumab	2017	CheckMate 040	NA	ORR: 20%	Second line, advanced HCC with or without hepatitis B/C	Adrenal insufficiency, diarrhea, hepatitis, infusion hypersensitivity, and acute kidney injury (1 each)	^47^
	PD-L1 VEGF	Atezolizumab Bevacizumab	2018	NCT02715531	NA	Confirmed partial response: 62%	First-line, advanced or metastatic	Hypertension (19%), autoimmune encephalitis, mental status change and intra-abdominal hemorrhage (8%)	^48^
Gastric	PD-1	Pembrolizumab	2018	Keynote 059	NA	ORR: 11.6% CR: 2.3%	Recurrent, locally advanced or metastatic; failed two prior lines	Hypothyroidism (8.9%), hyperthyroidism (3.5%), colitis (2.3%), pneumonitis (1.9%), death (0.8%- acute kidney injury, pleural effusion)	^75^

*PD-1* programmed death- 1, *CTLA4* cytotoxic T-lymphocyte-associated protein 4, *PD-L1* programmed death ligand-1, *VEGF* vascular endothelial growth factor, *MSI* microsatellite instability, *MSS* microsatellite stable, *ORR* objective response rate, *DCR* disease control rate, *PFS* progression-free survival, *CR* complete response

## Oncolytic viruses

Virus-based cancer treatments - sometimes referred to as virotherapy - have been promising, as adjuncts to immune based therapy options. Oncolytic viruses (OVs) inherently infect, replicate within, and lyse cancer cells while sparing normal cells. Growing body of literature suggests that OVs offer a combination of tumor-specific cell lysis together with immune stimulation, therefore acting as potential in situ tumor vaccines or immunotherapeutic agents.^51^ The OV-associated inflammatory response is optimal for antigen presentation and helps to reveal hidden tumor antigens. Dying cancer cell releases tumor antigens that are recognized by the immune system.^52^ To date, though a number of viruses are being evaluated as potential treatments for cancer in clinical trials, only one OV - genetically modified form of herpes virus called Talimogene laherparepvec (T-VEC) for treating metastatic melanoma - has been approved by the FDA.^53^

**Fig. 2:**
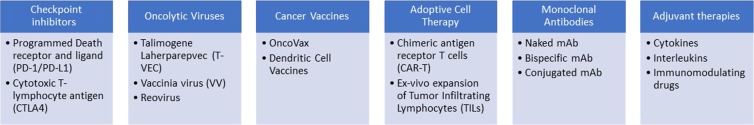
Overview of immunotherapeutic modalities

Several trials have been performed to test the appositeness of OVs in CRC management. Biweekly intravenous administration of Pexa-Vec (JX-594), an oncolytic and immunotherapeutic vaccinia virus (VV), in CRC has been shown safe and well-tolerated.^2^ Pexa-Vec is currently being tested combined with durvalumab, an anti-PD-L1 agent, in phase I and with tremelimumab, anti-CTLA-4 antibody, in phase II in patients with refractory metastatic CRC (NCT03206073). In another study, VV’s combination with oxaliplatin or SN-38 has been shown to be increasing median survival in mice and synergistic cell killing and causing S phase arrest in cultured CRC cell lines.^54^ Enadenotucirev (formerly known as ColoAd1), a novel group B Ad11p/Ad3 chimeric adenovirus, in combination with PD-1 checkpoint inhibitor nivolumab is being tested as phase I dose-escalation study (NCT02636036). LOAd703, an immunostimulatory oncolytic adenovirus has been investigated as single agent in phase I/II trial of patients with CRC. Treatment of CRC stem cells with oncolytic herpes simplex virus in preclinical models has shown enhanced cytotoxic effect.^55,56^

Reovirus, a naturally occurring and ubiquitous double-stranded RNA OV - commonly infected in mammals, including humans and mice, asymptomatically - has intrinsic preference for replication in KRAS mutant cells causing apoptosis.^57,58^ Replication happens in the cytoplasm of infected cells and culminates in the formation of crystalline arrays of progeny virions within viral inclusions. Additionally, reovirus can be useful to trigger immune system to kill cancer cells. Reovirus serotype 3 - Dearing Strain (Reolysin) – has been studied in phase I in combination with FOLFIRI and bevacizumab, an anti-VEGF-A agent, in FOLFIRI naive patients with KRAS mutant metastatic CRC (NCT01274624). In a Phase 1b study, it was tested along with chemotherapy and pembrolizumab, anti-PD-1 antibody, in patients with advanced (unresectable or metastatic) histologically confirmed pancreatic adenocarcinoma (MAP).^59^ The combination therapy showed manageable safety profiles and antitumor activity in previously treated MAP patients.

## Vaccine development

Immunotherapy has opened avenues to develop treatment strategies with both prophylactic and therapeutic implications. Figure 2 depicts the various immunotherapeutic modalities currently available in practice. Vaccines work on the principle of artificial immunity where the immune system is primed by introduction of foreign antigen, to which an immune response is elicited, and thus immune memory is created.^60^ Vaccines against non-viral tumors have mainly targeted differentiation antigens, cancer testis antigens, and overexpressed neo-antigens.

A phase III trial was performed as early as 1999 to study OncoVax, a patient-specific vaccine composed of metabolically-active, sterile, irradiated, and non-tumorigenic autologous colon cancer cells. The study compared changes in overall and progression-free survival by the addition of the vaccine to surgery compared to surgery alone, in patients with stage II/III CRC. While OncoVAX had a major impact on stage II disease with a significantly longer recurrence-free period and 61% risk reduction for recurrences, no significant changes were found in overall survival or stage III disease.^61^

There are numerous trials underway to identify and establish an effective vaccine in various gastrointestinal malignancies (Table 2). However, development of cancer vaccines has been challenging as the interaction of tumor and immune system is a dynamic, unremitting relationship. Evolution of a clone that has mutated or deleted the target antigen could become a resistance pathway of major clinical concern.^62,63^ Negative selection in the thymus against normal nonmutated antigens severely limits the ability to generate high avidity anti-cancer T cells. Such depletion can impair their antitumor activity and limit tumor elimination. Additionally, concerns about ballooning expenditures for new medical technologies certainly apply to these individually manufactured immunologically derived anticancer vaccines.

**Table 2:** A tabular compilation of the ongoing clinical trials along with the list of therapeutic agents, the targets, the clinical settings, phase and relevant comparator arm

	Therapeutic Agent	Target	Clinical Setting	Phase	Comparator arm	Identifier
Cancer vaccine	Messenger RNA (mRNA)-Based Personalized Cancer Vaccine	Neoantigens Expressed by the Autologous Cancer	Metastatic gastrointestinal cancer	I/II	NA	NCT03480152
	Peptide loaded dendritic cell vaccine	Long peptides and minimal epitopes from defined neoantigens or highly expressed mutations in tumor suppressor or driver genes	Melanoma Gastrointestinal Breast Ovarian Pancreatic	II	NA	NCT03300843
	Surgery and OncoVax	Sterile, live but non-dividing tumor cells administered as vaccine	Stage II Colon CA	III	Surgery	NCT02448173
	OBI-833/OBI-821	Globo H hexasaccharide 1 (Globo H) antigen conjugated to DT-CRM197, a non-toxic, mutated form of diphtheria toxin (DT)	Metastatic Gastric Metastatic Breast Metastatic Colorectal Metastatic Lung	I	NA	NCT02310464
Adoptive T cell therapy	Peripheral Blood Lymphocytes Transduced with a Murine T-Cell Receptor Recognizing the G12V Variant of Mutated RAS in HLA-A*1101 Patients	KRAS G12V molecule on the surface of tumors.	Pancreatic Gastric Colon Rectal	I/II	NA	NCT03190941
	Cytokine-induced killer cell immunotherapy along with radical surgery and adjuvant chemotherapy		Stage III CRC	III	Radical surgery and adjuvant chemotherapy	NCT02280278
	Cryosurgery and natural killer (NK) cell immunotherapy		Advanced esophageal cancer	I/II	Cryosurgery	NCT02843581
	Radiation Therapy and Peptide Specific CTL Therapy	Neoantigen peptide specific cytotoxic T lymphocytes (CTL)	unresectable advanced esophageal cancer	II	NA	NCT03011255
Combination Immunotherapy	Nivolumab + Ipilimumab	PD-1 + CTLA4	Cholangiocarcinoma/ duodenal carcinoma Neuroendocrine tumors Rare Gynecological tumors	II	NA	NCT02923934
	Oral cobimetinib with intravenous (IV) atezolizumab and bevacizumab	MEK + PD-L1 + VEGF	Metastatic colorectal cancer	Ib	NA	NCT02876224

## Adoptive T-cell therapy

Genetic engineering has allowed for ex vivo customization and expansion of autologous T-cells. In the CAR-T cell therapy, autologous T cells are extracted and transduced with a gene that encodes a chimeric antigen receptor (CAR) to direct the patient’s T cells against the tumor cells. These CAR-T cells are then expanded in a production facility and finally infused back to the patient. As a result, recognition of a specific cell surface antigen activates T-cell response independently of MHC recognition. This research has been most extensive in hematologic malignancies and the first CAR-T cell therapy, Tisagenlecleucel, was approved in Aug 2017, for relapsed refractory B-cell Acute Lymphoblastic Leukemia (B-ALL).^64^ There are numerous translational studies underway to identify a consistent antigen to serve as a target for the CAR-T cell therapy to be expanded to solid tumors as well. (Table 2)

Another form of adoptive T-cell therapy is ex-vivo expansion of tumor-infiltrating lymphocytes (TILs). TILs, as the name suggests, are immune cells such as Dendritic cells and Natural Killer cells isolated from the tumor tissue and thus recognize the tumor antigens. These are cultured ex-vivo with lymphokines such as interleukin-2 and then re-infused into the patient. The theory is that the immune cells are exhausted by the tumor microenvironment and the ex-vivo treatment allows them to be reintroduced at higher doses, by which they can overcome any tolerance by the tumor. While this form of therapy has been studied in various cancers such as melanoma, cholangiocarcinoma and cervical cancer, it has not yet received FDA approval in any cancer.^65–67^ A major limitation of this therapy is the unreliability of TIL extraction and expansion.

## Monoclonal antibodies

The addition of biologic agents (i.e., bevacizumab [humanized monoclonal antibody to vascular endothelial growth factor], cetuximab [chimeric] and panitumumab [fully human; monoclonal antibodies to epidermal growth factor receptor]) have led to modest improvements in survival. For example, with bevacizumab, addition to fluoropyrimidine-based chemotherapy regimens has led to statistically significant increases in median progression-free survival (PFS) and overall survival (OS). In one pooled analysis, PFS was found to be 9.1 vs. 6.9 months [*P* < .0001], and OS was 19.8 vs. 17.6 months [*P* = 0.0034]^68^ with and without bevacizumab, respectively. Bioengineering has allowed for creation of **“bispecific mAbs”** (bsAb or BiTE), a more recent addition to this class of drugs. Here, two mAbs with different antigen targets are fused together to increase specificity and allow for recruitment of immune cells directly to the site of tumor. A well-known example is blinatumomab, a drug used to treat Acute Lymphoblastic Leukemia.^8^ A BiTE antibody targeting the cetuximab binding site on EGFR has shown safety and efficacy in primates for *KRAS* and *BRAF* mutated CRC.^69^ An advantage of bsAb is that it can halt synergistic regulation by different receptor systems in a complex network of signaling pathways, by concurrently binding to two or more receptors.^70^

Newer mAbs such as LY3022855 are being designed, with novel targets such as Colony-stimulating factor 1 receptor (CSF1R), also known as macrophage colony-stimulating factor receptor (M-CSFR). CSF1R is a cell-surface receptor for its ligands, colony-stimulating factor 1 (CSF1) and IL-34.^71^ Increased CSF1 expression is implicated in tumor progression and metastasis, which is associated with poor prognosis in some cancers.^72^

## Future directions

It is evident that with the paradigm shift in oncology therapeutics, the field has consistently moved away from the cookie-cutter approach. Personalized medicine has taken the frontier where a tumor, its treatment and prognosis are defined by the genetic/epigenetic profile, protein expression and genetic mutations rather than a TNM stage. Tissue agnostic drug approvals are on the rise. It can be well perceived that there is an urgent need to identify additional biomarkers and cancer pathways, discern tumor heterogeneity at the molecular level, determine variability in cancer type and stage and have deeper insight into the underlying immunosuppressive mechanism of cancer to find the much-needed cure. Advances in preclinical knowledge and application of this at the bedside are quintessential for optimal outcomes.
